# Doxycycline vs Hydroxychloroquine + Azithromycin in the Management of COVID-19 Patients: An Open-Label Randomized Clinical Trial in Sub-Saharan Africa (DOXYCOV)

**DOI:** 10.7759/cureus.45619

**Published:** 2023-09-20

**Authors:** Eugene Sobngwi, Sylvain Zemsi, Magellan Guewo, Jean-Claude Katte, Charles Kouanfack, Liliane Mfeukeu, Armel Zemsi, Yves Wasnyo, Antoinette Ntsama Assiga, Arnaud Ndi Manga, Joelle Sobngwi-Tambekou, William Ngatchou, Charlotte Moussi Omgba, Jean-Claude Mbanya, Pierre Ongolo Zogo, Pierre Joseph Fouda

**Affiliations:** 1 Internal Medicine, Yaoundé Central Hospital, Yaoundé, CMR; 2 Faculty of Medicine and Biomedical Sciences, University of Yaoundé 1, Yaoundé, CMR; 3 Research, RSD (Research Science and Development) Institute, Yaoundé, CMR; 4 The Biotechnology Center, University of Yaoundé 1, Yaoundé, CMR; 5 Faculty of Medicine and Biomedical Sciences, University of Yaoundé 1, Yaounde, CMR; 6 Faculty of Medicine and Pharmaceutical Sciences, University of Dschang, Dschang, CMR; 7 Internal Medicine, Yaoundé Central Hospital, Yaounde, CMR; 8 Internal Medicine, Yaoundé Central Hosptial, Yaounde, CMR; 9 Research and Development, RSD (Research Science and Development) Institute, Yaoundé, CMR; 10 Hypertension Clinic, Erasme University Hospital, Brussels, BEL; 11 Regional Delegation of Public Health, Ministry of Public Health, Yaoundé, CMR; 12 Center for the Development of Good Practices in Health, Yaoundé Central Hospital, Yaoundé, CMR

**Keywords:** sub-saharan africa, mild covid-19, azithromycin, hydroxychloroquine, doxycycline

## Abstract

Objective

We aimed to compare the safety and efficacy of a doxycycline-based regimen against Cameroon National Standard Guidelines (hydroxychloroquine plus azithromycin) for the treatment of mild symptomatic COVID-19.

Methods

We conducted an open-label, randomized, non-inferiority trial in Cameroon comparing doxycycline 100 mg, twice daily for seven days versus hydroxychloroquine 400 mg daily for five days and azithromycin 500 mg at day 1 and 250 mg from day 2 through 5 in mild COVID-19 patients. Clinical recovery, biological parameters, and adverse events were assessed. The primary outcome was the proportion of clinical recovery on days 3, 10, and 30. Non-inferiority was determined by the clinical recovery rate between protocols with a 20-percentage points margin.

Results

One hundred and ninety-four participants underwent randomization and were treated either with doxycycline (n = 97) or hydroxychloroquine-azithromycin (n = 97). On day 3, 74/92 (80.4%) participants on doxycycline versus 77/95 (81.1%) on hydroxychloroquine-azithromycin-based protocols were asymptomatic (p = 0.91). On day 10, 88/92 (95.7%) participants on doxycycline versus 93/95 (97.9%) on hydroxychloroquine-azithromycin were asymptomatic (p = 0.44). On day 30, all participants were asymptomatic. The severe acute respiratory syndrome coronavirus 2 (SARS-CoV2) polymerase chain reaction (PCR) test was negative on day 10 in 60/92 (65.2%) participants who were assigned to doxycycline and in 63/95 (66.3%) participants who were assigned to hydroxychloroquine-azithromycin. None of the participants were admitted for worsening of the disease after treatment initiation.

Conclusion

Doxycycline 100 mg twice daily for seven days proved to be safe and non-inferior in terms of efficacy when compared to hydroxychloroquine-azithromycin for preventing clinical worsening of mild symptomatic or asymptomatic COVID-19 and achieving virological suppression.

## Introduction

Since the outbreak of COVID-19, no confirmed successful targeted treatment has emerged [1] for mild or moderate forms of disease. Nonetheless, symptomatic treatments and several therapeutic agents have demonstrated some clinical efficacy in clinical trials [2], including remdesivir, tocilicizumab, and convalescent plasma. The combination of hydroxychloroquine and azithromycin initially gained widespread use appearing in many national therapeutic guidelines mostly in Africa [1], which has a past history of extensive use of chloroquine for malaria treatment and prophylaxis. On the basis of in vitro evaluation against the severe acute respiratory syndrome coronavirus 1 (SARS-CoV-1) and SARS-CoV-2 and observational data [3], hydroxychloroquine and azithromycin remain the mainstay treatment of mild to moderate COVID-19 in many sub-Saharan African countries [4], despite concerns about possible adverse drug reactions when used in combination at high doses. In the absence of approved treatment for SARS-CoV-2, numerous alternative empirical treatments are being suggested. These comprise contemporary and traditional pharmacopeia with known anti-inflammatory and antiviral properties.

Doxycycline is a derivative of tetracycline that possesses broad antimicrobial and anti-inflammatory activities [5,6]. Doxycycline was FDA-approved as an antibiotic in 1967 and to this day remains in the antibiotic arsenal for diverse clinical use [7]. It has demonstrated antimicrobial, antiparasitic, and antiviral properties in several studies [8], including against some coronaviruses. Moreover, it is generally well accepted and tolerated in clinical settings [8].

The main objective of treating mild and moderate forms of COVID-19 is to prevent adverse progression to severe presentation requiring admission and/or intensive care. We thus aimed to compare the safety and efficacy of doxycycline to that of the national standard guidelines (hydroxychloroquine plus azithromycin) for the treatment of mild symptomatic and asymptomatic COVID-19 patients receiving ambulatory care in Cameroon to prevent the disease from evolving to more severe forms potentially requiring admission.

This article was previously posted to the medRxiv preprint server in July 2021.

## Materials and methods

Study design

The trial was an open-label, randomized, non-inferiority trial to evaluate the safety and efficacy of doxycycline therapy versus Cameroon National Standard therapy (hydroxychloroquine + azithromycin) in ambulatory patients with mild symptomatic or asymptomatic COVID-19. 

Study site and period

The trial was conducted at the Yaoundé Central Hospital Coronavirus Treatment Center between March 16 and April 09, 2021. The Yaoundé Central Hospital is a second-category hospital in the Cameroon health pyramid. It is a decentralized structure of the Ministry of Public Health and has a capacity of about 650 beds for all specialties and a considerable human resource of more than 600 employees. During the COVID-19 pandemic, it was one of the main hospitals that took care of infected patients.

Study participants

Eligible participants were 18 years of age or older, who had COVID-19 infection confirmed by SARS-CoV-2 real-time polymerase chain reaction (RT-PCR) test and were asymptomatic or were mildly symptomatic with an SpO2 >94%. An asymptomatic patient was defined as a patient with a positive COVID-19 test with no symptoms [9]. A mild symptomatic patient was defined as a patient with a positive COVID-19 test with any of the following symptoms, including fever less than 103°F (39.4°C), fatigue, cough, sore throat, headache, muscle pain, malaise, nausea, vomiting, loss of taste and smell, lack of appetite, and nasal congestion [9]. The patients were excluded if they were pregnant, breastfeeding, had currently taken trial study medication, had any contraindication to study medication, had known severe cardiac disease, renal or liver insufficiency, respiratory rate ≥30/min, and BP ˂90/60 mmHg. All participants gave written informed consent before any trial procedures were performed. 

Randomization, allocation concealment, and blinding

The participants were randomly assigned, in a 1:1 ratio, to receive hydroxychloroquine + azithromycin combination or doxycycline combination in a central procedure performed before the start of the trial. Randomization codes were computer-generated, using the Random allocation software (Randomization Main) with a one-block size. Randomly generated treatment allocation was given to clinical trial medical doctors within sealed opaque envelopes. Once a patient had consented to enter the trial, the corresponding envelope was opened, and the patient was then offered the allocated treatment regimen. 

Study procedures and intervention

Patients assigned to the study arm received a fixed-dose combination of doxycycline (100 mg PO) twice daily for seven days. Patients assigned to the National guideline received hydroxychloroquine 400 mg daily for five days; azithromycin 500 mg on day 1 and 250 mg from day 2 to day 5. Concomitant medications included vitamin C 1000 mg once daily for five days, and oral zinc 20 mg once daily for five days in both arms. 

Data collection and management

Trial visits were scheduled for the baseline, day 3, day 10, and day 30 to evaluate the safety of the trial drugs. RT-PCR control tests to evaluate the efficacy of the treatment were performed on day 10. Blood samples were collected following good laboratory practice by venipuncture of the brachial vein in a 5 ml dry tube without a tourniquet. These samples were collected at baseline and day 10 for full blood count and glycemia.

Study outcomes

The primary endpoint was the rate of clinical recovery determined by the percentage of participants who became or remained asymptomatic. Key secondary endpoints included the percentage of participants requiring hospitalization due to worsening symptoms, as well as the proportion of participants displaying a negative SARS-CoV-2 PCR test. Changes in full blood count and glycemia from the initial measurement were also evaluated. All endpoints were assessed on day 3, day 10, and day 30 in relation to the baseline. Safety assessments involved monitoring of adverse events and mortality, occurring from the first administration of the study treatment to the final follow-up visit on day 30 in accordance with the protocol. 

Sample size and statistical analyses

The study was designed with 80% power to test the primary hypotheses, employing a non-inferiority margin of 20% and type 1 error of 0.05 giving a minimum sample size of 194 participants. We computed a two-sided 95% confidence interval (CI) to assess the disparity between treatments with regard to the proportion of patients achieving clinical recovery, using the unstratified approach outlined by Miettinen and Numinen [10]. 

The condition to define the non-inferiority between doxycycline and hydroxychloroquine-azithromycin-based protocols was established if the lower limit of the two-sided 95% CI for the treatment difference (doxycycline minus hydroxychloroquine-azithromycin) was greater than -20%. Additionally, a p-value was calculated for the corresponding one-sided non-inferiority hypothesis test. Efficacy, safety, and patient-reported outcomes were analyzed within the Intention-to-treat population. All statistical analyses were carried out using SPSS version 23.0 (IBM Corp, Armonk, NY).

Data Safety Monitoring Board

An independent data and safety monitoring committee revised safety and efficacy data during the trial. The data safety monitoring committee was able to pause the trial or give suggestions on potential safety issues to improve the study design and intervention.

## Results

Trial population

**Figure 1:**
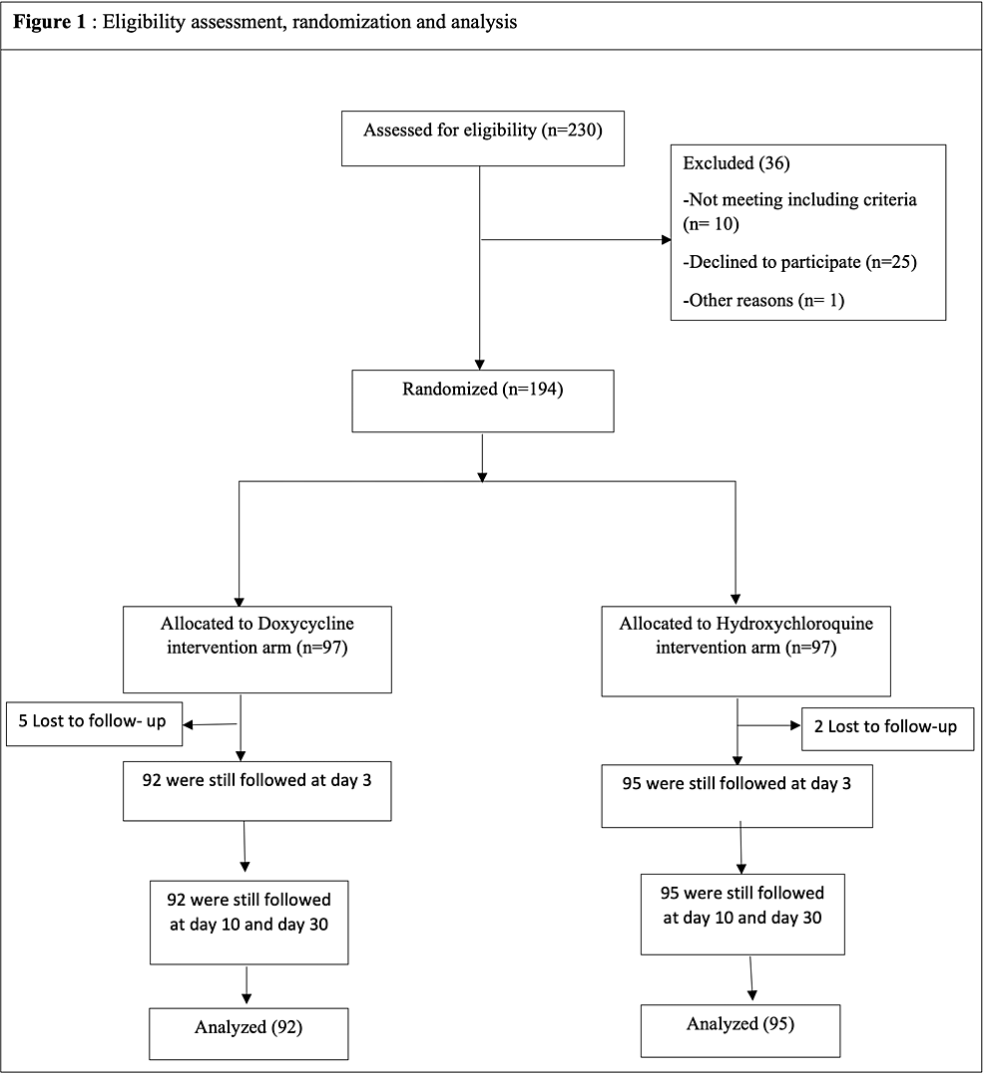
Eligibility assessment, randomization, and analysis.

From the 194 participants who underwent randomization, at least one dose of the assigned treatment was administered (Figure 1). Baseline and disease characteristics of participants are shown in Table 1. The mean age was 39 years and 47.6% of participants were women. 42.2% (79/187) of the analyzed participants were mildly symptomatic at baseline (42/92 on the doxycycline baseline regimen versus 37/95 on the hydroxychloroquine-azithromycin arm treatment) (Table 1). The most common symptoms were fatigue, muscle or joint pain, anosmia, cough, headache, and chill.

**Table 1:** Baseline characteristics HTN: hypertension; SD: standard deviation; RBC: red blood cell; HGB: hemoglobin; HCT: hematocrit; MCV: mean corpuscular volume; WBC: white blood cell; LYM: lymphocyte; NEUT: neutrophil; PLT: platelet; DRx: doxycycline; HCTa: hydroxychloroquine + azithromycin; FBC: full blood count.

Baseline characteristics	DRx (N = 92)	HCTa (N = 95)	Total (N = 187)	p-Value
Age - years, mean (SD)	38 (±13)	39 (±14)	39 (±13)	0.76
		Number (%)		
Sex female - n (%)	49 (53.3)	40 (42.1)	89 (47.6)	0.14
Comorbidities				
Asthma (%)	2 (2.2)	1 (1.1)	3 (1.6)	
HIV (%)	0 (0.0)	1 (1.1)	1 (0.5)	0.55
Treated HTN (%)	1 (1.1)	1 (1.1)	2 (1.1)	
No comorbidities (%)	89 (96.7)	92 (96.7)	181 (96.8)	
Baseline symptoms				
Asymptomatic	50 (54.3)	58 (61.1)	108 (57.8)	0.55
Mild illness	42 (45.7)	37 (38.9)	79 (42.2)	
Baseline FBC		Mean (±SD)		
RBC tera/l	5.0 (±1.0)	5.0 (±1.1)	5.0 (±1.0)	0.95
HGB g/l	13.1 (±2.9)	13.1 (±3.6)	13.1 (±3.3)	0.93
HCT %	45.6 (±11.2)	45.8 (±12.5)	45.7 (±11.8)	0.92
MCV fl	91.8 (±7.9)	89.1 (±12.2)	87.9 (±8.5)	0.11
WBC giga/l	5.3 (±2.1)	5.0 (±1.8)	5.2 (±2.0)	0.53
LYM giga/l	2.2 (±0.8)	2.1 (±0.8)	2.1 (±0.9)	0.36
NEUT giga/l	3.0 (±1.6)	2.6 (±1.1)	2.8 (±1.4)	0.08
PLT giga/l	204 (±75.7)	202 (±72.9)	203 (±74.1)	0.95
Baseline glycemia				
Glycemia	0.94 (±0.2)	1.0 (±0.6)	0.98 (±0.3)	0.32

Clinical and patient-reported outcomes

Doxycycline was non-inferior to hydroxychloroquine-azithromycin for the primary endpoint (Table 2). On day 3, the clinical recovery rate was at 80.4% (74/92) for doxycycline and 81.1 % (77/95) for hydroxychloroquine-azithromycin [difference (95%) CI, 0.6% (-0.1, 0.1)]. On day 10, the clinical recovery rate was at 95.7% (88/92) for doxycycline and 97.9 % (93/95) for hydroxychloroquine-azithromycin [difference (95%) CI, 2.2% (-0.07, 0.03)]. On day 30, all the participants were asymptomatic.

**Table 2:** Clinical recovery rate by protocol visit DRx: doxycycline; HCTa: hydroxychloroquine + azithromycin.

Clinical status	DRx n (%)	HCTa n (%)	CI	p-Value
Day3				
Asymptomatic	74 (80.4)	77 (81.1)	[-0.1, 0.1]	0.91
Mild Illness	18 (19.6)	18 (18.9)		
Day 10				
Asymptomatic	88 (95.7)	93 (97.9)	[-0.07, 0.03]	0.44
Mild Illness	4 (4.3)	2 (2.1)		
Day30				
Asymptomatic	92 (100)	95 (100)		

SARS-CoV2 PCR testing

The rate of SARS-CoV2 PCR negative test on day 10 was 65.2% (60/92) in the doxycycline arm and 66.3% (63/95) in the hydroxychloroquine + azithromycin arm. The difference between treatment groups was 1.1 percentage points (95% CI, [-0.15, 0.13]; p > 0.05) (Table 3).

**Table 3:** Percentage of negative SARS-CoV-2 RT-PCR test on day 10 DRx: doxycycline; HCTa: hydroxychloroquine + azithromycin; SARS-CoV2, severe acute respiratory syndrome coronavirus 2; RT-PCR, real-time polymerase chain reaction.

Assessment visit day 10	DRx n (%)	HCTa n (%)	Difference, % (95% CI)	p-Value
Negative PCR-COVID-19	60 (65.2)	63 (66.3)	1.1 [-0.15, 0.13]	>0.05

Adverse events

No major adverse event was reported. None of our participants was admitted for worsening of the disease after treatment initiation. There was no difference between treatment arms regarding changes in full blood count and glycemia from baseline (Table 4).

**Table 4:** Biological parameters of participants on day 10 SD: standard deviation; RBC: red blood cell; HGB: hemoglobin; HCT: hematocrit; MCV: mean corpuscular volume; WBC: white blood cell; LYM: lymphocyte; NEUT: neutrophil; PLT: platelet; DRx: doxycycline; HCTa: hydroxychloroquine + azithromycin.

Biological parameters	Day 10		CI	p-Value
	DRx	HCTa		
	Mean (SD)			
RBC tera/l (SD)	4.6 (±0.8)	4.8 (±0.5)	[-0.4, 0.1]	0.21
HGB g/l (SD)	12.2 (±3.8)	12.2 (±1.7)	[-1.1, 1.2]	0.93
HCT % (SD)	41.9 (±6.8)	41.3 (±8.8)	[-2.5, 3.7]	0.69
MCV fl (SD)	90.3 (±7.3)	88.9 (±7.8)	[-1.5, 4.4]	0.33
WBC giga/l (SD)	5.2 (±2.4)	5.0 (±1.3)	[-0.6, 0.9]	0.64
LYM giga/l (SD)	2.1 (±0.8)	2.2 (±0.7)	[-0.4, 0.1]	0.32
NEUT giga/l (SD)	3.0 (±1.3)	2.8 (±1.3)	[-0.3, 0.7]	0.46
PLT giga/l (SD)	247 (±72.6)	230 (±75.8)	[-12.0, 46.0]	0.25
Glycemia (SD)	0.99 (±0.1)	0.95 (±0.2)	[-0.06, 0.15]	0.41

## Discussion

Since the outbreak of COVID-19 in December 2019, the world has been going through waves of the pandemic of widely variable levels of clinical severity. Up to 80% of people affected develop an asymptomatic or mildly symptomatic disease. It remains unclear whether a specific treatment is required for mild COVID-19. However, taking into consideration the risk of adverse progression toward severe and critical forms of the disease, several therapeutic approaches are proposed worldwide to prevent the need for hospitalization and admission to critical care units.

In early 2020, a small series of COVID-19 patients treated in France with hydroxychloroquine showed a rapid decline in SARS-CoV-2 viral load compared with controls, which seemed further improved by the addition of azithromycin [11]. However, methodological flaws were reported, limiting the interpretation of the results [12], in addition to a few other conflicting trials on the use of hydroxychloroquine, and its possible cardiac adverse events [3]. Nevertheless, some countries, especially in sub-Saharan Africa, still recommend hydroxychloroquine-azithromycin as the reference treatment in the management of COVID-19 patients [13]. The development of vaccines is expected to drastically reduce hospital and intensive care admissions as well as deaths. In the meantime, many other treatment options for mild and moderate COVID-19 were investigated to mitigate progression toward severe disease, hospitalization, or death with variable efficacy, including mostly drugs with antiviral and anti-inflammatory effects [14,15]. 

In this randomized, open-label trial, we compared doxycycline with hydroxychloroquine-azithromycin for the treatment of ambulatory patients with mild symptomatic or asymptomatic COVID-19. None of the participants progressed to severe disease nor required hospital admission, and we showed the non-inferiority of a seven-day course of doxycycline to a five-day course of hydroxychloroquine-azithromycin in terms of clinical recovery rate, virological suppression, and safety profile. No major adverse event was recorded possibly due to the young age of the study population, the safe dosage used in our participant (400 mg hydroxychloroquine and 250 mg azithromycin), and prior exposures to the study drugs for malaria, and in accordance with our preliminary safety study [16].

Our results support the observation by Mohammud M Alam et al. in 2021 in the United States of America, where they evaluated the clinical outcomes of early treatment with doxycycline for 89 high-risk COVID-19 patients in long-term care facilities, and found that early treatment with doxycycline for high-risk patients with moderate-to-severe COVID-19 infections in non-hospital settings is associated with early clinical recovery, decreased hospitalization, and decreased mortality [17]. The present trial was not placebo-controlled, but the >95% cure rate on day 10 contrasts with the 70% reported in placebo groups on day 14 in another trial [18].

While the study population was relatively young with limited comorbidities, it reflects the general population of Cameroon and most sub-Saharan countries. In 2019, the Cameroon Demographic Health Survey estimated that 60% of the population is aged 20-40 years, and only 14% of the population is above 40 [19]. In the earlier stages of the COVID-19 pandemic, older age was considered among the important factors associated with clinical severity [20]; however, recent reports indicated, in more than 3000 adults ages 18-34 who contracted COVID-19 and became sick enough [20], to require hospital care, 21% ended up in intensive care, 10% were placed on a breathing machine, and 2.7% died. Young adults are therefore a major target population for interventions that would reduce progression to severity to mitigate the public health and economic effects of the pandemic. 

There are several limitations to this study. Primarily, the absence of a placebo arm within the study to assess the significance of the two treatments being investigated for COVID-19 management. However, it is important to acknowledge that during the period of conducting this research, obtaining ethical approval from the Cameroon National Ethics Committee to conduct a study wherein certain participants would not receive any treatment after testing positive for COVID-19 would have presented significant ethical challenges and constraints. Other limitations of this study include the absence of follow-up of participants at the end of the trial to find out long-term adverse drug effects. Finally, although the study recorded a manageable rate of loss to follow-up, a comprehensible outcome assessment for all initially enrolled participants could not be achieved.

## Conclusions

In conclusion, we did not observe any significant difference in terms of safety and efficacy between doxycycline and hydroxychloroquine-azithromycin for the management of mild COVID-19. Future research should consider including a cohort of participants who do not receive any treatment. The group would be monitored for the same study timeline, with a focus on assessing identical outcomes, and would help evaluate the necessity of these two regimens in the management of COVID-19 in a sub-Saharan population. A post-study follow-up might also be necessary for all the participants to evaluate the impact of COVID long and drug adverse effects.
